# Factors predicts skin ulcer following coronary artery bypass

**Published:** 2014

**Authors:** F Sabzi, R Faraji

**Affiliations:** * Department of Cardiovascular Surgery, Imam Ali Heart Center, Kermanshah University of Medical Sciences, Kermanshah, Iran

**Keywords:** cardiac surgery, complication, skin injury

## Abstract

The number of off-pump coronary artery surgery procedures in high-risk patients such as renal failure, hepatic failure and in anticoagulant drug using patients is increasing. The associated co morbidity and repeated use of electrocautery in postoperative bleeding, caused a susceptibility of patients to pressure or electrocautery ulcers. During a period of three years, 1400 off-pump coronary artery bypass surgery were performed in our center. Of these patients, 20 (A group) suffered from electrocautery sore (ES) and 40 (B group) had pressure sore (PS). These patients were compared with respect to variables such as age, hypertension, hypercholesterolemia, operating time, smoking, opium using, diabetes, weight, sex, respiratory failure, renal failure, and cerebrovascular accident, intra aortic balloon pump using, inotropic drug using by x2 or t test, according to categorical or continuous variables consequently. Electrocautery sore and pressure ulcer as dependence variables and others variables with p value less than 0.1 entered a multivariable logistic regression model and odd ratio of significant variables were obtained. These two groups of patients were different with respect to variables such as age, sex, respiratory failure and cerebrovascular accident and, in the logistic regression model, two factors predicted pressure sore, respiratory failure and cerebrovascular accident, but the only factor that was significant in predicting electrocautery sore in multiple logistic regression analysis was postoperative bleeding. Results of this study revealed that pressure sore is a patient dependent complication in contrast with the electrocautery sore, which is related to technical or device faults and to experience and care of operating room staff.

**Abbreviations:** electrocautery sore = ES, pressure sore = PS, electrocautery ulcer = EU, pressure ulcer = PU, activated clotting time = ACT

## Introduction

Despite the great attention and concern that have been paid by surgeons, operating room staff, and engineering personnel, patients continue to suffer incidental skin sore in the operating room. Such injuries can prolong hospital stay, and increase hospital costs. Electrocautery devices units are frequently concerned about intraoperative skin sores. In many cases, the injury may be a fault in the electrosurgical unit or its connection or malfunction of that device. The other differential diagnosis of cautery sore includes bed sore, an adverse warfarin reaction, or a disease process that develops in the area in which a device was applied such as reaction to povidone iodine or other antiseptic solutions [**1**-**3**]. Depending on the time of occurrences and the location of the burn, the equipment, devices, and solutions that may have to be inspected include, electrocautery device and its components, including hand piece electrodes, earth electrodes, cables, and conductive gel , hypo/hyperthermia units with associated blankets and patient rectal or nasopharyngeal temperature probes, heating pads, heat lamps, radiant warmers, diathermy units, transcutaneous oxygen and carbon dioxide electrodes, pulse oximeter probes, bispectral index electrodes, intra-aortic balloon pumps and their accessories include electrodes, cables, tube, tourniquets, monitors with associated cables, electrodes, and probes, cardiopulmonary bypass equipment, operating room tables, anesthesia masks and tubing, prepping and antiseptic agents, ointments, and linens and allergic reaction to adhesives, electrode gel, ointment, and skin prep solution like povidone iodine and deconex [**4**,**5**]. But, diagram seems to be the most important device that predisposes the patient to intraoperative burn in the electrocautery unit [**6**-**8**]. The average output to the hand piece electrode is of 400 watts, but may exceed to 800 watts if the tip of the hand piece has debris. The power output of the electrocautery unit is purposely concentrated in the active electrode. Usually, the body skin has a high resistance, in the average of 4000 ohms to the usual current flow of 400 or 600 watts [**9**]. This article presents the risk factors associated with electrocautery burn and pressure sore along with literature reviews for conducting a thorough electrocautery burn study.

## Methods

Between December 2009 and December 2001, 1400 OPCAB were performed in our center. Of these 1400 OPCAB, 60 patients had postoperative sore. The preoperative characteristics of the patients are listed in **Table 2**. The study was approved in our center and the patients’ consents were obtained. Preoperative and postoperative variables were retrospectively collected and entered into a database form. 20 patients had electrocautery ulcer (EU) (group A) and 40 patients had pressure ulcer (PU) (group B). These two groups were compared with respect to categorical or continuous variables by t test or x2 test. Significant variables with P-value less than 0.1 entered in logistic regression model and odd ratio of significant variables were obtained.

**Table 2:** Demographic and intraoperative variables compared by t test and x2

Variable	Pressure ulcer	Electrocautery ulcer	P-value
Hypercholesterolemia	34%	26%	Ns
Packed red cell consumption	2±.2 unit	1.3±.12	Ns
Weight	72.6±5.1	75.1±4.6	Ns
Time spend end on operating table	3.1 hour	3.23 hour	Ns
Smoking	38.7%	34.4%	Ns
Age (mean±SD)	66±12	54±11	<0.05
Diabetes	26.6%	12.5%	<0.05
Sex (female %)	45%	38%	Ns
Hypertension	41%	31%	Ns
Opium using	31%	28%	Ns
Ejection fraction	48±5	50±7	Ns

**Definition**

**Operation technique:**

Saphenous vein grafts and left internal mammary artery were always anastomosed to the left anterior descending artery. Intraoperative monitoring of patients undergoing off-pump CABG surgery typically included a combination of the following arterial pressure monitoring, electrocardiographic monitoring and sometimes the transesophageal echocardiography to detect well motion abnormality or valve incompetence, Anesthesia management included the use of short acting anesthetic agents, pain control, and right lung ventilation to improve access and reduce lung movement by inflation and deflation of the left lung. Other immobilization techniques included the use of intraoperative esmolol and preoperative intravenous inderal (1-2 mg) to induce bradycardia and help reduce the technical difficulty of performing surgery on a beating heart. 100 mg/kg Heparin was administered to keep the activated clotting time (ACT) between 200-400 seconds. The two stabilization techniques involved the use of either a suction or compression device, suction stabilization lifted the epicardium and pulled the tissue target to the immobilized target area. Before the anastomosis, the target coronary artery was temporarily occluded proximally and distally by fine bulldog clamps or looped 5/ 0 Vilene suture. If the systolic blood pressure was reduced to 70 mmHg or less, a inter luminal shunt was used. Phenylephrine (1-2 mg) was intravenously administered to the patients whose hemodynamic status was unsuitable to keep the blood pressure between 70-90 mmHg.

## Results

The demographic and clinical data are summarized in **Table 2**. Of the 60 patients, 66% were men; the mean age was 66±12 and 54 ±11 in pressure ulcer and electrocautery ulcer patients respectively. The mean left ventricle ejection fraction was 0.45±0.15 (Range, 15% to 55%). Of the 1400 patients, 17 died in the hospital (1.2%) but no death occurred in the electrocautery ulcer group. In the PU group, one patient died after prolonged hospital stay. The most common place in cautery sore was the sacrum, followed by the occipital, and the heels. In the univariable analysis, two groups were different with respect to age, diabetes, hypertension, respiratory failure, renal failure, weight and stroke. The PU groups were older, diabetic and hypertensive compared to the cautery ulcer group. In the multivariate regression analysis, the predicting factors following PU were strokes, hypertension, inotropic drug usage, IABP insertion and respiratory failure, but the most important factor for the prediction of ES was the re-exploration due to bleeding. The technical analysis demonstrated that 20 cases of cautery induced ulcer, in 5 cases, poor quality of earth paddle (china made) caused a subsidiary way, created by the patients’ perspiration, and, in 6 cases, an incorrect connection of earth paddle electrode, caused burn; 1 burn occurred after moisture under the paddle electrode, eight burns occurred during surgery while irrigating fluid or disinfecting solution or leaking blood from sternal sutures created alternate current pathways, one burn was chemical, after skin preparation with Deconex solution, and in one case, the cause of the burn was the use of a warming bag with hot water. The last two cases were excluded from the study. Preoperatively, PU patients had a significantly higher incidence of hypertension, smoking, opium use, hypercholesterolemia, as compared with CU, as well as a significantly higher incidence of diabetes and a lower weight (**Table 2**). However, there were no significant differences according to the ejection fraction and gender between the two groups. 40 patients developed a total of 43 pressure ulcers, and three developed more than one pressure ulcer. Twenty patients developed cautery ulcers, as did 12 (60%) of the men and 40% of woman (P < 0.001). 55% of pressure ulcers were rated as grade 2, 30% were grade 3, and 15% were grade 4 (**Table 4**).

**Table 4:** Electro cautery sore grade and location

Location	Number of sore	Grade 1	Grade 2	Grade 3	Grade 4
Occipital	5 (25%)	-	3	2	-
Back and shoulder	3 (15%)	-	1	1	1
Arm	1 (5%)	-	1	-	-
Hip	-	-	-	-	-
Sacrum	7 (35%)	-	3	2	2
Ischial tuberosity	-	-	-	-	-
Leg	1 (5%)	-	1	-	-
Heel	3 (15%)	-	2	1	-
	-	-	-	-	-

Seven ulcers (17.7%) progressed to upper grade, during the first 10 days observation period. These included 4 grades I ulcers to grade II, two to grade III, and one to grade IV. One patient with respiratory failure and prolonged intubation and a grade 4 pressure ulcer died. The most common locations for the pressure ulcers were the sacrum (67.5%), sacrum and buttock (17.5%), buttock (10%) and occipital (5%). The most common site of electrocautery ulcers were the sacrum (40%), occipital (25%), back-shoulder (15%), heel (15%), arm (5%) and leg (5%). Pressure ulcer patients were older (66±12years) than the electrocautery ulcer patients (54 ± 11 years) (P < 0.05). Factors associated with pressure ulcer development among our patients, as identified in the multivariate regression analysis, were stroke and respiratory failure. The multivariate regression analysis revealed that the re-exploration for bleeding was significant in predicting the electro cautery ulcer. Nutritional state, mobility, friction and shear stress were not evaluated in our study. Further associated variables in univariate analysis were diabetes, age, respiratory failure, stroke, renal failure, IABP usage, inotropic drug usage and hypertension. Intra and postoperatively, there were no significant differences in the time spent on the operating table, packed cell consumption, between patients who developed pressure ulcers and those who developed cautery ulcer.

## Discussion

Electrocautery burns, as results of exposure of the patient's skin to a high-frequency electrical circuit, with modern electrosurgical equipments that are able to selectively cauterize tissues by scattering current density as it leaves the patient's body, are very rare [**10**]. In Igner et al. study, the overall incidence of recognized aberrant electrocautery burns was between 1 and 2 patients per thousand operations [**11**]. Batigg et al. have shown that direct contact burns in the operative field have resulted from an imprecise active electrode use [**12**]. Stienke et al. found that the improper placement or attachment of the grounding electrode could lead to burns at the site of indifferent electrode attachment [**13**]. Heating and evaporations of the collected fluid by electrocautery device can cause skin burn as it happened in one of our patients. Vedovato et al. [**14**] have attributed this complication to the heating solution that pooled underneath the patients by both the active and the indifferent electrode. Isager et al. [**15**] stated that alternate intraoperative circuits could be generated by an operative device, such as the bed table or hand holder metal grip contacting the patient's body, leading to thermal injury at sites of contact, remote from the operative field. Such subsidiary circuits caused “capacitive coupling” burns and occurred when the current preferentially passed from the active electrode through a grounding site other than the paddle electrode. In our cases, there were 5 occipital thermal burns as results of capacitive coupling burn. In two cases in our series, the Bispectral index lead was connected to the patients’ head and, in three cases, severe sweating of scalp and wetness of occipital area in connection to an insulated surgical table, caused occipital small burn. Parker et al. reported that alternate circuits could cause a temperature probe insertion site burn in the esophagus and late stenosis [**16**]. One of our patients was admitted with non-carcinomatous fibrous stricture in the upper segment of the esophagus. In another patient in our series, the rectal probe that was settled for an on-pump CABG remained in the rectum and caused a rectal burn that was confirmed by rectoscopy. In our study, the most common site of pressure ulcer was the sacrum (67.5%), followed by sacrum & buttock (17.5%), buttock (10%) and occipital area (10%) consequently (**Table 3**). 

**Table 3:** Variables predicting pressure ulcer following off-pump CABG

Variable	Β	S.E	Wald	Df	Sig	Odd.ratio	CI
Respiratory failure	5.7	2.6	4.6	1	.03	33	1.7-6337
Stroke	.206	.099	4.2	1	.04	1.9	1-1.04

Sometimes, during the electrocautery use, there is an interference in a monitoring signal or a parasite noted in the monitor screen plate. This condition needs concern and immediate investigation. If in doubt, it should be checked carefully. Unusually, if an increased power is necessary to obtain an adequate cautery effect, the earth-plate circuit or loop is probably faulty and precautions are recommended. In contrast to the thermal sore, bedsore has a different pathophysiological mechanism. The fundamental factor in the development of PU is the unrelieved pressure when tissues are compressed between 2 firm surfaces. It is logical to know that the sacrum area is the most predisposing place for developments of both the pressure and the thermal sore [**17**]. The incidence of the sacral sore with cautery and pressure in our study was of 35% and 67.5% respectively. In an univariate analysis, the following factors were different between two groups: inotropic drug use, IABP insertion, hypertension, respiratory failure, renal failure, stroke and diabetes (**Table 1**). 

**Table 1:** Comparison of the patient’s characteristics between two groups

Variables	Electro cautery sore	Pressure sore	P-value
Location of sore	Occipital (25%)	Occipital (5%)	<0.05
	Heel (15%)	Heel (0%)	<0.05
	Trunk (15%)	Trunk (0%)	<0.05
	Sacrum (40%)	Sacrum (67.5%)	<0.05
	Sacrum & buttock (0%)	Sacrum & buttock (17.5%)	<0.05
	Buttock (0%)	Buttock (10%)	>0.05
	Arm (5%)	Arm (0%)	>0.05
Respiratory failure	(2%)	(21%)	<0.05
Renal Failure	(1%)	(7%)	<0.05
Cerebrovascular accident	(0%)	(10%)	<0.05
Inotropic drug usage	5%	30%	<0.05
IABP usage	2%	7%	<0.05

The above-mentioned risk factors denote a low cardiac output, tissue acidosis and ischemia that predispose to pressure sore. This most commonly occurs over the bony protrusion on the body, where soft tissue was compressed between an external surface such as the bed and an internal unyielding surface of the bone. The common occurrence of both ulcers in our study denotes that this hypothesis is logical. Tissue tolerance for pressure reflects the ability of skin and supporting structures to tolerate the effect of pressure. The closing capillary pressure was of 30 mmHg and when pressures on internal tissues exceed the closing capillary pressure (30-32 mmHg) for more than 120 minutes, circulation was compromised and tissue anoxia and death were ensued. The positional changing has not been considered properly by the ICU staff and, in most instances, this time frame (120 minute) for the prevention of capillary pressure closing was missed [**18**]. In the multivariate regression analysis, the only factor predicting the cautery sore was the re-exploration for bleeding. In an emergency condition, like tamponade and hypotension, especially at night, when professional staff will not be present, the ignorance of the inexperienced staff, time limitation for mediastinal exploration, mediastinal blood soaking of the patients’ body, urgency and improper ground pad placement, an increased incidence of cautery sore formation would be caused [**19**]. The amount and duration of the reaction hyperemia depends on the skin texture condition and is increased in the ischemic part. One of the most important factors in intrinsic factor is age. To determine the impact of age on PU development, Papantonio [**20**] and associates, involved 136 adults in their study, who were scheduled for cardiac surgery, due to multiple risk factors, they found age as a significant risk factor for the development of pressure ulcer. In our study, patients with ES were younger compared to PU patients, however, in the univariate analysis, these two group of patients were different with respect to age, diabetes and hypertension (**Table 2**). In the multivariable logistic regression, age was not a significant factor in the development of ES. We believe that the cause of this difference, related to the basic mechanism of these two types of sore, hypertension, diabetes and older age, causes skin microvascular change and predispose the skin and subcutaneous tissue to complication of immobility and its sequels. These three factors do not have a direct effect on the creation of CU but aging, exhaustion of subcutaneous tissue and musculature atrophy predispose to the malfunction of earth paddle by the mechanism of poor conductivity of exhausted subcutaneous tissue. However, there is not any report about the change of skin conductivity in diabetes patients. 

## Conclusion

The complete evaluation of a skin injury includes the consideration of all the possible device faults or body preparation solution interactions. It must also consider the possibility that the injury may be a pressure sore electrocautery ulcer, thermal injury or reaction to drugs or antiseptic solution. The medical engineer and the surgical staff team in the operating room must make sure that all the possibilities and probabilities are considered in the evaluation of a sore and that the surgeon and staff involved in the incident is questioned. Only then will it be possible to develop effective preventive measures.

**Acknowledgments**

The authors would like to acknowledge the kind efforts made by the statistical department and nurses at Imam Ali Hospital, Kermanshah University of Medical Sciences, Kermanshah, Iran.
